# Carotid atherosclerotic lesion analysis in 3D based on distance encoding in mesh representation

**DOI:** 10.1007/s11548-025-03464-4

**Published:** 2025-06-30

**Authors:** Hinrich Rahlfs, Markus Hüllebrand, Sebastian Schmitter, Christoph Strecker, Andreas Harloff, Anja Hennemuth

**Affiliations:** 1 Institute of Computer-Assisted Cardiovascular Medicine, Deutsches Herzzentrum der Charité, Berlin, Germany; 2 Fraunhofer MEVIS, Bremen, Germany; 3 DZHK (German Centre for Cardiovascular Research), Partner Site, Berlin, Germany; 4 Physikalisch-Technische Bundesanstalt (PTB), Berlin, Germany; 5 Department of Neurology and Neurophysiology, Faculty of Medicine, Medical Center-University of Freiburg, Freiburg, Germany

**Keywords:** Carotid artery, Vessel wall, MRI, Atherosclerosis

## Abstract

**Purpose:**

The purpose of this study is to support stroke risk analysis, evaluation of therapy effectiveness, and lesion progression through a comprehensive assessment of carotid atherosclerotic lesions in 3D based on automatic segmentation and visualization of quantitative parameters.

**Methods:**

We propose a novel method for extracting the pathologically thickened regions from 3D vessel wall segmentations using distance encoding on the inner and outer wall mesh to enable atherosclerotic lesion analysis. A case-specific and constant threshold was evaluated and applied to extract lesions from a dataset of 202 T1-weighted black-blood MRI scans of subjects with up to 50% stenosis. Applied to baseline and one-year follow-up data, the method supports detailed morphology analysis over time, including volume quantification, aided by improved visualization via mesh unfolding. The extracted region was also used to analyze the signal intensity distribution within the lesion region.

**Results:**

We successfully extracted lesion regions from 297 carotid arteries, capturing a wide range of shapes with volumes ranging from 3.61 to $996.9~{\textrm{mm}}^{3}$. The use of a constant threshold of 1.6 mm showed an intraclass correlation of 0.861 for the lesion volume and a median average surface distance of 0.594 mm in the validation set.

**Conclusion:**

The proposed method enables the extraction of lesion meshes from 3D vessel wall segmentation masks, enabling a correspondence between baseline and one-year follow-up examinations. Unfolding the lesion meshes enhances visualization, while the mesh-based analysis allows quantification of morphologic parameters and an analysis of the signal intensities in the lesion region.

## Introduction

**Fig. 1:**
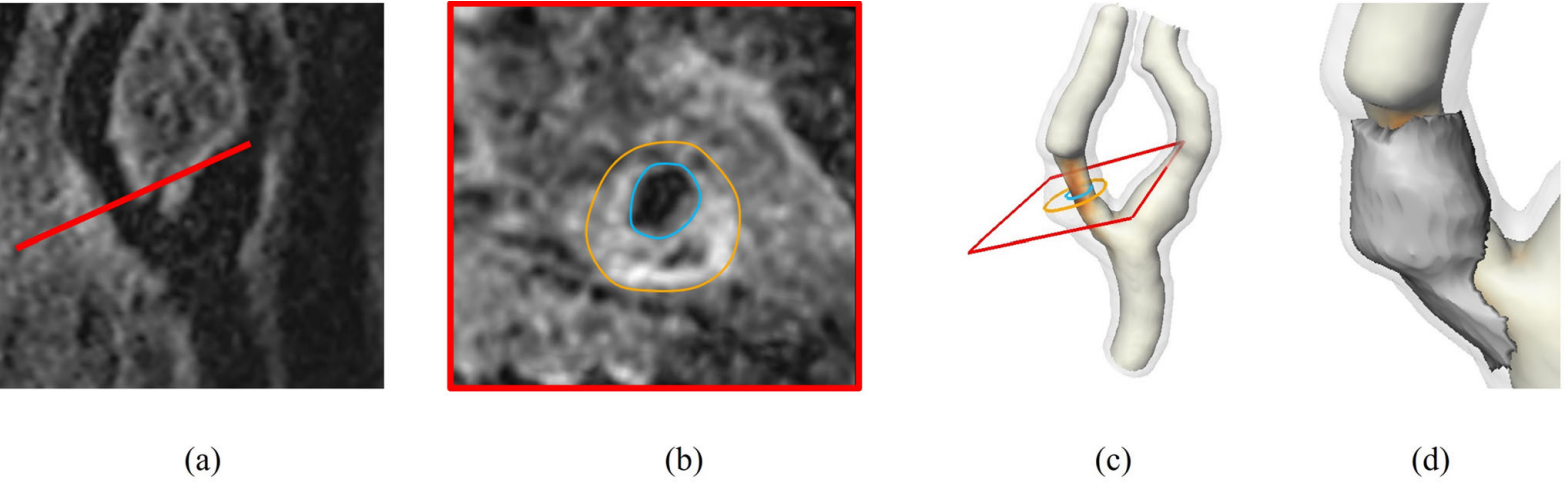
**a** View of an atherosclerotic carotid artery in T1-weighted 3D BB-MRI with cross-section MPR. **b** Segmented 2D cross section of the carotid artery which can be used for the computation of wall area and maximum wall thickness. **c** 3D segmentation of the carotid wall with the VWT color-coded on the lumen surface. **d** Lesion extracted from the 3D segmentation which can be used for the quantification of lesion volume, surface, and compactness

Atherosclerosis of the carotid artery and the resulting internal carotid artery stenosis are a major risk factor for stroke [1], which is a leading cause of disability and mortality worldwide [2]. Atherosclerotic lesions develop over time, and magnetic resonance imaging (MRI) enables noninvasive assessment [3].

Quantitative progression parameters are required for therapy monitoring, stroke risk assessment, and analysis of lesion progression. In clinical studies, manually segmented 2D cross-sectional images are used to extract parameters such as vessel wall thickness (VWT) [4–6] and normalized wall area [7, 8]. This method is sensitive to the chosen 2D cross-section location and orientation (Fig. 1a, b) and does not include information about the extent of the vessel wall thickening along the vessel course. The volume of the thickened vessel areas has the potential to be a better parameter for stroke risk assessment if the automatic quantification is implemented [9, 10].

**Fig. 2:**
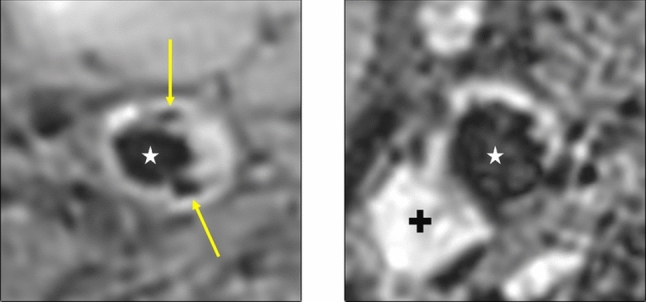
T1-weighted BB-MRI of two carotid arteries. The lumen is marked with a white star. On the left, calcification is present. The calcification has low signal intensity. Calcified areas are marked with a yellow arrow. On the right, intraplaque hemorrhage was detected. Hemorrhage has high signal intensity in T1-weighted BB-MRI and is marked with a black plus

In addition to the morphologic assessment of the atherosclerotic lesion, the vessel wall composition is relevant for atherosclerotic lesion classification and stroke risk assessment. Differing signal intensities in multi-contrast MRI allow a differentiation between fibrous tissue, lipid or necrotic cores, calcification, intraplaque hemorrhage, and thrombus [3]. Examples of a calcified plaque and intraplaque hemorrhage in T1-weighted black-blood MRI (BB-MRI) are shown in Fig. 2 and highlight the relevance of intensity analysis for a lesion classification.

Direct automatic 3D segmentation of plaque components in 3D BB-MRI is challenging due to limited annotated datasets and high variability in plaque composition and morphology. Further, intermediate lesions (Type III according to the AHA classification) show as diffuse thickening in MRI and a plaque is not necessarily visible [3]. While automatic segmentation of plaque in CTA was proven to be possible, it often requires manual refinement [11]. Recent advancements enabled reliable 3D segmentation of the carotid artery wall in MRI [12–15]. We propose to extract the area of the atherosclerotic lesion based on the VWT between the inner and outer vessel wall computed from the vessel wall segmentation. Specifically, we define a “lesion region” as the part of the vessel wall with increased thickness and use this to assess the lesion morphology and the signal intensity distribution within the lesion region.

Vessel maps have been developed for a broad variety of applications, including the visualization of the VWT in the carotid artery bifurcation [16, 17], as well as providing an overview of the carotid arteries’ lumen radius and enabling navigation for cross-section analysis [18]. Eulzer et al. [19] proposed a method that is able to automatically cut and unfold an arbitrary vessel tree and demonstrated that the method works for the carotid artery, including more than one bifurcation. Choi et al. [17] enabled improved area preservation and therefore enabled comparability between examinations. While these techniques found solutions for the cutting of the carotid artery in one or multiple bifurcations, the extraction of the lesion region allows us to create a vascular map that does not require prior cutting or untangling. We show examples of baseline and follow-up examinations to qualitatively evaluate whether changes in the vessel wall thickness can be recognized using this easily accessible approach.

## Methods

### Data

We used a total of 212 T1-weighted black-blood (BB)-MRI scans of the neck region, covering the left and right carotid arteries close to the bifurcation. The scans were obtained using a Siemens Prisma 3T scanner with a T1-weighted 3D Turbo Spin Echo sequence (3D-SPACE) that incorporates fat saturation and dark blood preparation. Detailed descriptions of the data are provided by Strecker et al. [5, 6]. The scans were divided into a stenosis set and a healthy set.

The stenosis set consists of 202 BB-MRI scans. In total, 121 subjects were scanned in a baseline examination, and 81 of these subjects were scanned in a one-year follow-up examination. All subjects of the stenosis set were diagnosed with hypertension, each presenting at least one additional cardiovascular risk factor. Each subject had a plaque measuring $\ge$ 1.5 mm in the left and/or right carotid artery in ultrasound. All subjects have an ICA stenosis smaller than 50%, as defined by NASCET criteria [20]. The average age of subjects in the stenosis set was 70.7 years and the average body mass index (BMI) was $26.6\,\frac{\text {kg}}{\text {m}^{2}}$.

The healthy set consists of 10 BB-MRI scans of healthy subjects with an average age of 34.1 years and an average BMI of $24.0\,\frac{{\text {kg}}}{{\text {m}}^{2}}$. There was no follow-up examination performed for healthy subjects.

For the evaluation of different approaches and hyperparameters, a validation set was created. The inner and outer vessel walls, as well as the regions with increased VWT (if present), were manually segmented in 3D using CaroTo, an extension of the MEVISFlow software [21]. Due to the high effort required for manual 3D segmentation, the validation set only consists of eight randomly chosen MRI scans (16 carotid arteries) from the stenosis set and three randomly chosen MRI scans (six carotid arteries) from the healthy set. The eight subjects of the stenosis set had an average age of 67.9 years and an average BMI of $27.1\,\frac{\text {kg}}{\text {m}^{2}}$, and the three subjects of the healthy set had an average age of 32.7 years and an average BMI of $24.9\,\frac{\text {kg}}{\text {m}^{2}}$.

### Lesion mesh extraction

**Fig. 3:**
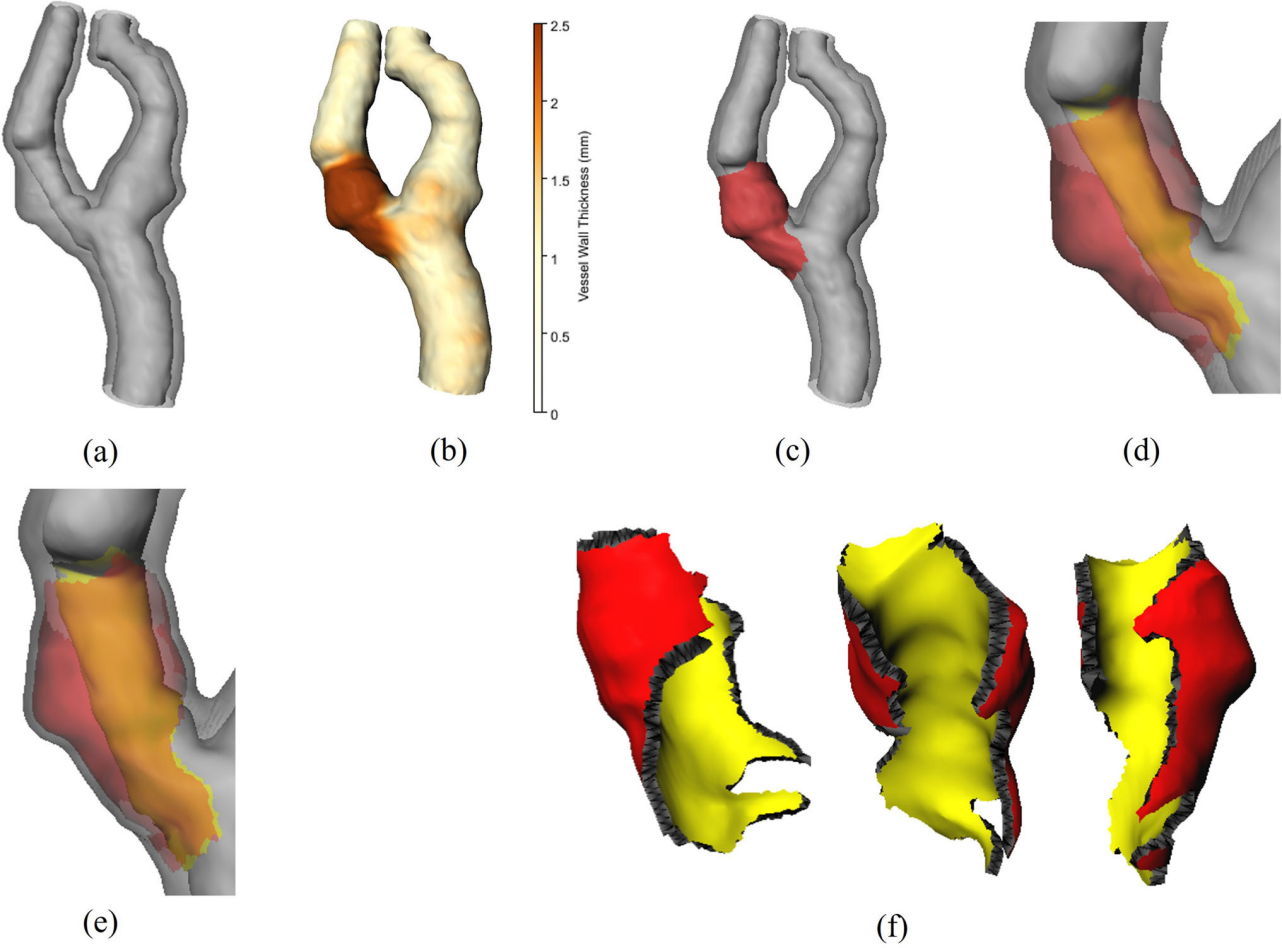
Process of lesion mesh extraction. **a** Inner and outer wall mesh created by the marching cubes algorithm [22] and smoothed with a windowed sinc filter. **b** Outer wall mesh color-coded with the distance to the inner wall mesh. **c** Lesion region (red) detected on the outer wall mesh. **d** Corresponding lesion region on the inner wall mesh (yellow). **e** Inner and outer border meshes, created by shifting all vertices of the lesion regions by half the mean VWT of the nonlesion vertices. **f** The created lesion mesh with the boundary mesh from different angles

***Vessel wall mesh creation***

The carotid artery wall and lumen were segmented using a 3D U-Net. It was trained on 3D pseudo-labels that were created using an auxiliary 2D U-Net as described by Rahlfs et al. [13]. The resulting voxel mask was transformed into triangle meshes using the marching cubes algorithm [22] on the lumen mask for the inner wall mesh and on the union of the lumen and wall mask to obtain the outer wall mesh. Smoothing was applied using a windowed sinc filter with a passband of 0.05 and a polynomial of degree 20. The result is shown in Fig. 3a.

***Lesion region extraction***

The lesion region was extracted using the distances between the vertices $X_{\text {outer}}$ of the outer wall and the inner wall mesh $M_{\text {inner}}$ (Fig. 3b). The distance between a point and the inner mesh $\text {dist}_{\text {mesh}}(x, M_{\text {inner}})$ is the minimal Euclidean distance from point $x$ to any triangle $T$ in $M_{\text {inner}}$, defined as:

1 $$\begin{aligned} \text {dist}_{\text {mesh}}(x, M_{\text {inner}}) = \min _{T \in M_{\text {inner}}} \text {dist}(x, T) \end{aligned}$$

The distance between a point and a triangle was computed using the ClosestPtPointTriangle algorithm proposed by Crister Ericson [23]. It utilizes the edges Voronoi regions for an efficient computation. The lesion vertices $X_{\text {lesion}}$ were then detected given the lesion threshold *lt*.

2 $$\begin{aligned} X_{\text {lesion}} = \{ x \in {X_{\text {outer}}} \mid \text {dist}_{\text {mesh}}(x, M_\text {inner}) > lt \} \end{aligned}$$

We defined the relevant lesion region as the submeshes with all face vertices in $X_{\text {lesion}}$. Submeshes with an area smaller than $10\,{\textrm{m}}^{2}$ were discarded. An example result is shown in Fig. 3c.

We evaluated two approaches for the threshold *lt*. Different global thresholds $lt\in \{1.4\,{\text {mm}}, 1.5\,{\text {mm}}, 1.6\,{\text {mm}}, 1.7\,{\text {mm}},$ $1.8\,{\text {mm}}, 1.9\,{\text {mm}}\}$ were evaluated. The value range was chosen based on Zhang et al. [24], who state that the mean VWT of the CCA measured with BB-MRI is 0.98 mm and the standard deviation is 0.2 mm.

The second approach was the use of a case-specific threshold. This threshold was computed iteratively using Eq. 3 and 4. As initialization, all outer vertices are normal vertices (Eq. 5). The threshold is computed by Eq. 4, and $X_{\text {normal}}$ is updated using the new threshold. The two steps are repeated until convergence. We evaluated this approach for $k\in \{2, 2.5, 3, 3.5, 4\}$.

3 $$\begin{aligned} {X_{\text {normal}}^{{\text {t}}+1}} = \{ x \in {X_{\text {outer}}} \mid \text {dist}_{\text {mesh}}(x, M_\text {inner}) \le {lt(X_{\text {normal}}^{\text {t}}}) \} \end{aligned}$$

where

4 $$\begin{aligned} {lt(X_{\text {normal}}^{\text {t}}})= &   {\mu (X_{\text {normal}}^{\text {t}}}) + {k*\sigma (X_{\text {normal}}^{\text {t}}}) \end{aligned}$$

5 $$\begin{aligned} {X_{\text {normal}}^{0}}= &   {X_{\text {outer}}} \end{aligned}$$

A vertex of the inner wall is classified as part of the inner lesion region if a ray, cast from the vertex along its normal, intersects the outer lesion region before any other part of the outer or inner wall. The inner lesion region is then defined as the submesh formed by these vertices. The result can be seen in Fig. 3d.

***Lesion mesh creation***

To include only the volume that was added due to increased VWT, the inner and outer lesion region vertices were shifted toward each other by half the mean VWT. This can either be 0.98 mm [24] if using the fixed threshold approach or the mean distances between $X_{\text {normal}}$ and $M_{\text {outer}}$ if using the case-specific approach. This leads to the inner and outer wall of the lesion, as shown in Fig. 3e.

As a next step, the borders of the inner and outer lesion meshes were stitched together as shown in Fig. 3f. To ensure that all lesion meshes are watertight, we used the MeshFix algorithm of Attene et al. [25].

### Vessel map visualization

Visualization of parameters on surfaces in 3D is not always intuitive, and surface unfolding can improve the parameter visualization [26, 27]. To improve the visualization, we unfold the lesion region extracted from the outer vessel wall and display the VWT on the unfolded 2D meshes. To unfold the meshes, we followed Eulzer et al. [19] and used the ARAP method [28] on the bijective mapping that was created with the LSCM algorithm [29].

### Geometric lesion parameters

The following parameters were derived from the extracted lesion meshes:

- Lesion Volume (*V*): The volume enclosed by the lesion mesh
- Lesion Surface Area (*A*): The area of the lesion mesh surface
- Compactness (*C*): Compactness of the lesion normalized to an ideal sphere
  6 $$\begin{aligned} C = \frac{36 \pi V^2}{A^3} \end{aligned}$$
- Maximum Extent: The maximum distance between two vertices of the mesh

### Intensity distribution within lesion mesh

To analyze the intensity distributions within the lesions, all voxels whose center points fall inside the lesion mesh were identified. The T1-weighted BB-MRI intensities corresponding to these voxels were extracted and used to construct a histogram.

### Ablation study on threshold method

For the choice of threshold method, we performed an ablation study on the different threshold settings. We extracted the lesion region for the 22 carotid arteries in the validation set and computed the median Dice coefficient (DC), median Hausdorff distance (HD), median symmetric average surface distance (ASD), and the intraclass correlation (ICC) for the volume, area, and compactness. For the ICC, the two-way mixed effects model, the single rater type, and the consistency definition [30] were used. For further analysis, we used the constant threshold of *lt*=1.6 mm based on the results shown in Table 1.

Given two watertight meshes $A$ and $B$, the Dice coefficient (DC) between $A$ and $B$ is computed as follows:

7 $$\begin{aligned} \text {DC}(A, B) ={\left\{ \begin{array}{ll} 1, &  \text {if } A \text { and } B \text { are both empty} \\ 0, &  \text {if exactly one of } A \text { or } B \text { is empty} \\ \frac{2 \cdot \text {vol}(A \cap B)}{\text {vol}(A) + \text {vol}(B)}, &  \text {otherwise} \end{array}\right. } \end{aligned}$$

where $\text {vol}(A)$ and $\text {vol}(B)$ denote the volumes of $A$ and $B$, and $\text {vol}(A \cap B)$ denotes the volume of their intersection.

The Hausdorff distance (HD) between A and B is computed as:

8 $$\begin{aligned}  &   \text {HD}(A, B) = \max (\text {HD}_s(A, B), \text {HD}_s(B,A)) \end{aligned}$$

9 $$\begin{aligned}  &   \text {HD}_s(A, B) ={\left\{ \begin{array}{ll} 0, &  \text {if } A \text { and } B \text { are both empty} \\ {\text {inf}}, &  \text {if exactly one of } A \text { or } B \text { is empty} \\ \max _{x \in X_A} {\text {dist}_{\text {mesh}}}(x, B), &  \text {otherwise} \end{array}\right. } \end{aligned}$$

The symmetric average surface distance (ASD) between A and B is computed as:

10 $$\begin{aligned}  &   \text {ASD}(A, B) = \frac{(\text {ASD}_s(A, B), \text {ASD}_s(B,A))}{2} \end{aligned}$$

11 $$\begin{aligned}  &   \text {ASD}_s(A, B) ={\left\{ \begin{array}{ll} 0, &  \text {if } A \text { and } B \text { are both empty} \\ {\text {inf}}, &  \text {if exactly one of } A \text { or } B \text { is empty} \\ {\text {mean}}_{x \in X_A} {\text {dist}_{\text {mesh}}}(x, B), & \text {otherwise} \end{array}\right. } \nonumber \\ \end{aligned}$$

## Results

### Ablation study on threshold method

Table 1 shows that the constant and variable threshold method can achieve a segmentation of the lesion region, with the constant threshold reaching higher DC and higher ICC, especially for the ICC of the area and the compactness. The following results were created using the constant lesions threshold $lt=1.6\,{\text {mm}}$.

**Table 1:** Ablation study on the influence of the constant and variable threshold with different parameters for the lesion threshold *lt* and the standard deviation factor *k*. For DC, HD, and ASD, the median value in mm is stated. ICC V,A,C stand for the ICC(3,1) of the lesion volume, area, and compactness

Method		DC	HD	ASD	ICC V	ICC A	ICC C
Constant	*lt* = 1.4 mm	0.612	5.432	**0**.**582**	0.864	0.663	0.407
Constant	*lt*= 1.5 mm	0.619	5.031	0.666	**0**.**874**	**0**.**756**	0.362
Constant	*lt*= 1.6 mm	**0**.**632**	**4**.**401**	0.594	0.861	0.748	**0**.**738**
Constant	*lt*= 1.7 mm	0.594	5.272	0.599	0.841	0.717	0.606
Constant	*lt*= 1.8 mm	0.562	4.510	0.767	0.815	0.661	0.500
Constant	*lt*= 1.9 mm	0.531	6.136	1.103	0.796	0.633	0.456
Variable	*k*= 2.0	0.596	11.737	1.245	0.867	0.594	−0.251
Variable	*k*= 2.5	0.558	6.829	1.086	0.808	0.683	0.145
Variable	*k*= 3.0	0.546	6.406	1.030	0.732	0.524	0.106
Variable	*k*= 3.5	0.517	6.973	1.161	0.688	0.443	0.218
Variable	*k*= 4.0	0.511	5.964	0.863	0.615	0.282	0.319

The bold values indicate the best performance: highest DC and lowest values for ACD, HD and ICC

### Results of baseline and follow-up examinations

**Fig. 4:**
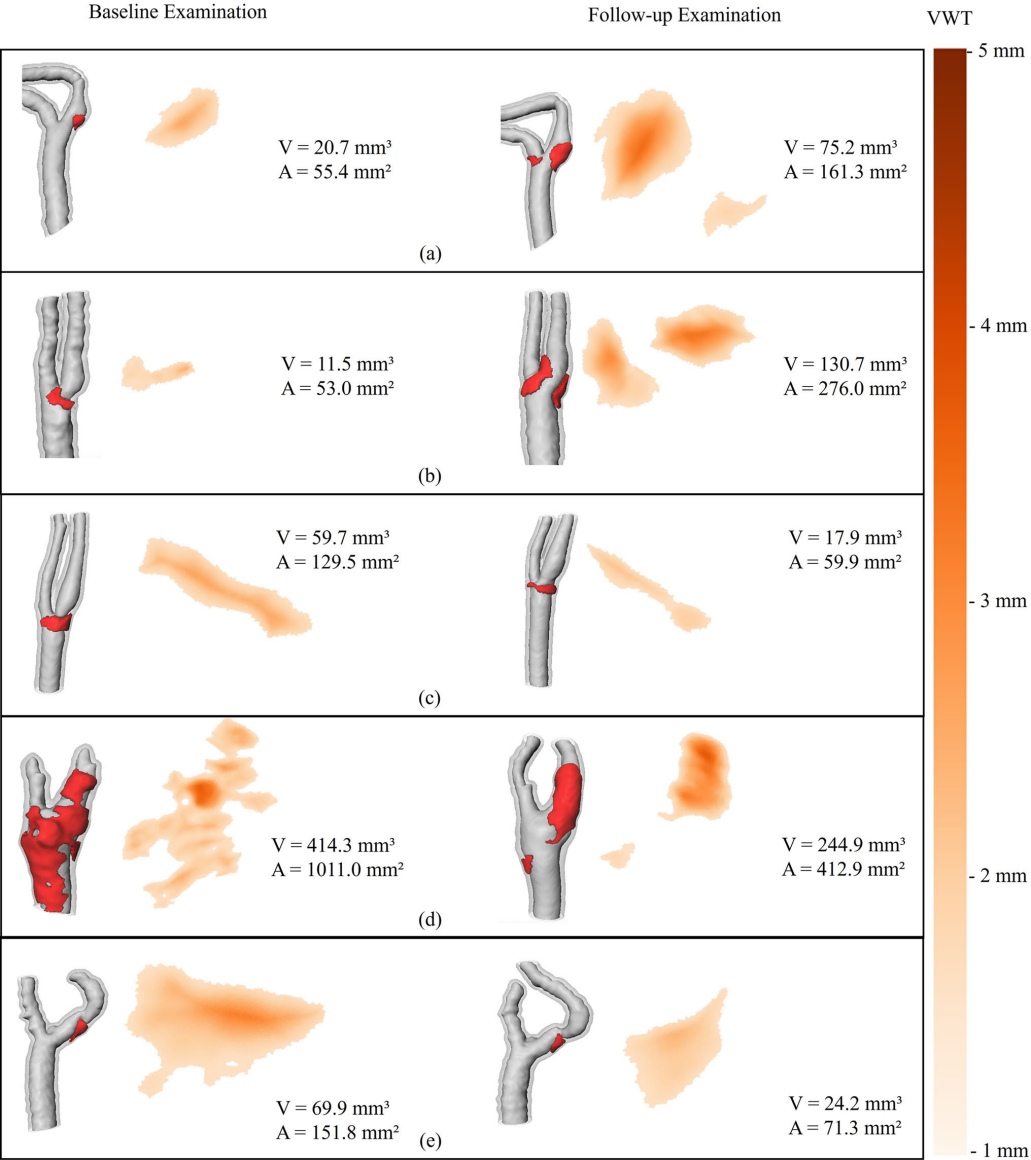
Lesion extraction using constant lesion threshold $lt=1.6\,{\text {mm}}$ on the baseline and 1-year follow-up scans of five carotid arteries. For each examination, the inner and outer wall meshes are shown on the left with the lesion mesh added in red. On the right, vessel map is shown color-coded with the vessel wall thickness (VWT). The zoom factor for an individual is constant, allowing a comparison between the baseline and follow-up examination

We chose five subjects with follow-up measurements for the qualitative evaluation and assessed the plausibility and interpretability of the quantitative and visual results. Figure 4 shows the inner wall mesh with the lesion mesh, the lesion mesh from a different angle, and the map visualization of the region with increased VWT, color-coded with the VWT. Figure 4a shows the results for a vessel with high tortuosity. The surface area (*A*), the VWT, and lesion volume (V) are increased in the follow-up examination. The second carotid artery (Fig. 4b) has an irregular, wavelike surface. This case also shows a strong growth in lesion area, volume, and VWT between baseline and follow-up. In (Fig. 4c), the extracted lesion in the follow-up examination is smaller. The lesion is extracted at the same position in both examinations. Figure 4d shows a carotid artery with a thickened wall for large parts of the CCA and ICA in the baseline examination. In the follow-up, the extracted lesion is localized in the ICA. The unfolded lesion region displays a growth of the area with high VWT. The last carotid artery (Fig. 4e) shows a lesion that is restricted to the ICA.

### Intensity histograms for plaque composition analysis

**Fig. 5:**
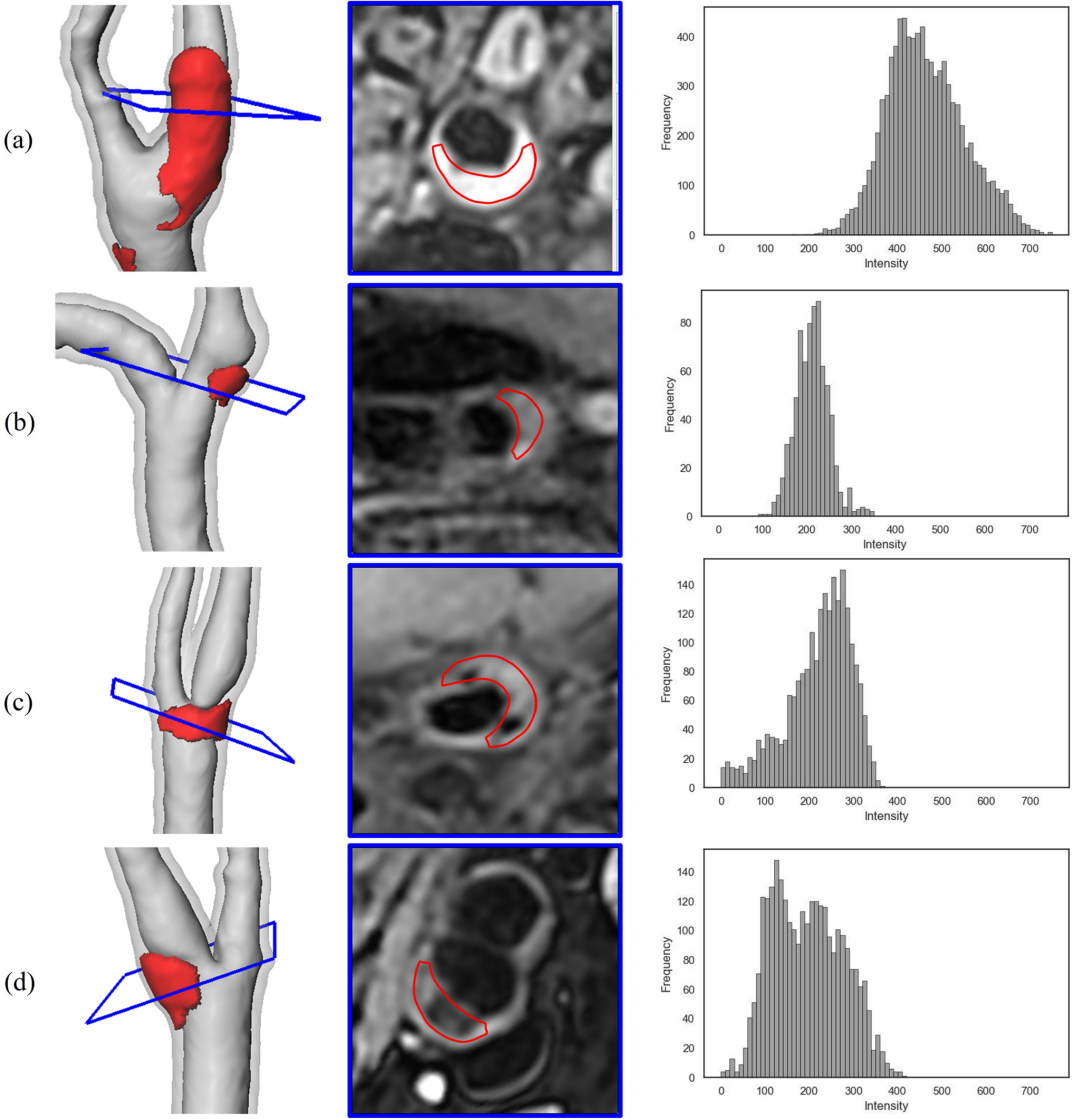
Analysis of T1-weighted MRI intensities within the lesion mesh. The lesion position (left), a 2D cross section of the T1-weighted MRI (middle), and the histogram of the intensities of all voxels inside the lesion mesh

Figure 5 shows the intensity distribution of the voxels inside the lesion mesh. The lesion in Fig. 5a shows hyperintense voxels, resulting in one peak in the histogram. The carotid artery wall below (Fig. 5b) shows two small hyperintensities, which are partly included in the lesion mesh. The corresponding histogram shows the hyperintensities as a small peak at intensity 325. Figure 5c shows a plaque with two calcifications represented as hypointense regions in the MRI images (see cross section), and the histogram shows a high skewness. Figure 5d shows a carotid artery wall with a plaque showing as a larger hypointense region. Correspondingly, the histogram shows two peaks.

### Lesion parameter distribution across all datasets

Using the global threshold $lt=1.6\,{\text {mm}}$, at least one region with increased VWT was extracted in 175 of the 202 MRI scans of the stenosis set. Looking at the individual arteries a lesion region was extracted from 297 of the 404 carotid arteries in the stenosis set.

**Fig. 6:**
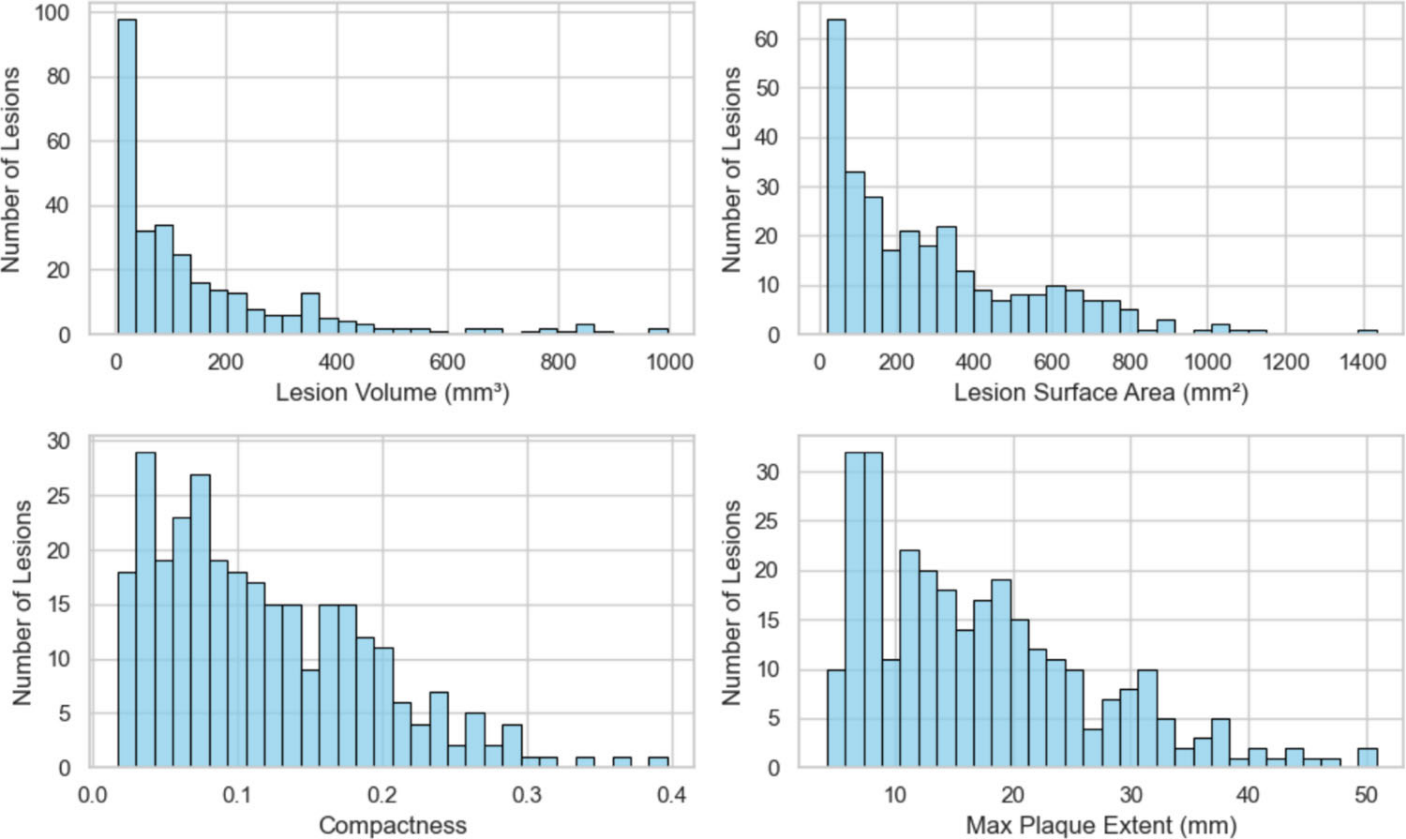
Distribution of lesion parameters. For the calculation of the parameters, the 297 lesion meshes were extracted with the constant threshold $lt=1.6\,{\text {mm}}$

Figure 6 shows the quantitative geometric parameters calculated using the lesion meshes. In total, 97 lesions have a lesion volume of less than $36.7\,{\text {mm}}^{3}$, and the maximum lesion volume is $996.9\,{\text {mm}}^{3}$. The average compactness of the lesion is 0.118, and the lesions can have an extent of up to 50.8 mm.

## Discussion

The ablation study showed that a constant threshold of $lt=1.6\,{\text {mm}}$ showed the highest median DC and the highest sum of ICC volume, ICC area, and ICC compactness. Therefore, this constant threshold is preferable. The proposed method achieved an ICC(3,1) of 0.86 for lesion volume and 0.75 for lesion area. Compared to the ICC of 0.84 reported for automatic assessment of VWT by Rahlfs et al. [31], the volume shows higher agreement, while the area shows lower agreement. Even though each dataset contains at least one plaque as measured with ultrasound, the method did not extract a lesion region in 13.4% of the datasets.

The mesh-based extraction approach has two advantages. It can extract and analyze a lesion with subvoxel resolution, and it allows a direct quantification of lesion features using established mesh algorithms. However, the inner and outer wall mesh has to be created with the marching cubes algorithm and a smoothing, which can introduce errors. A wall segmentation method that directly outputs a mesh could mitigate this issue. This could be achieved by utilizing the voxel2mesh architecture [32] which can be adapted for vessel tree segmentation [33].

***Dependence on segmentation***

**Fig. 7:**
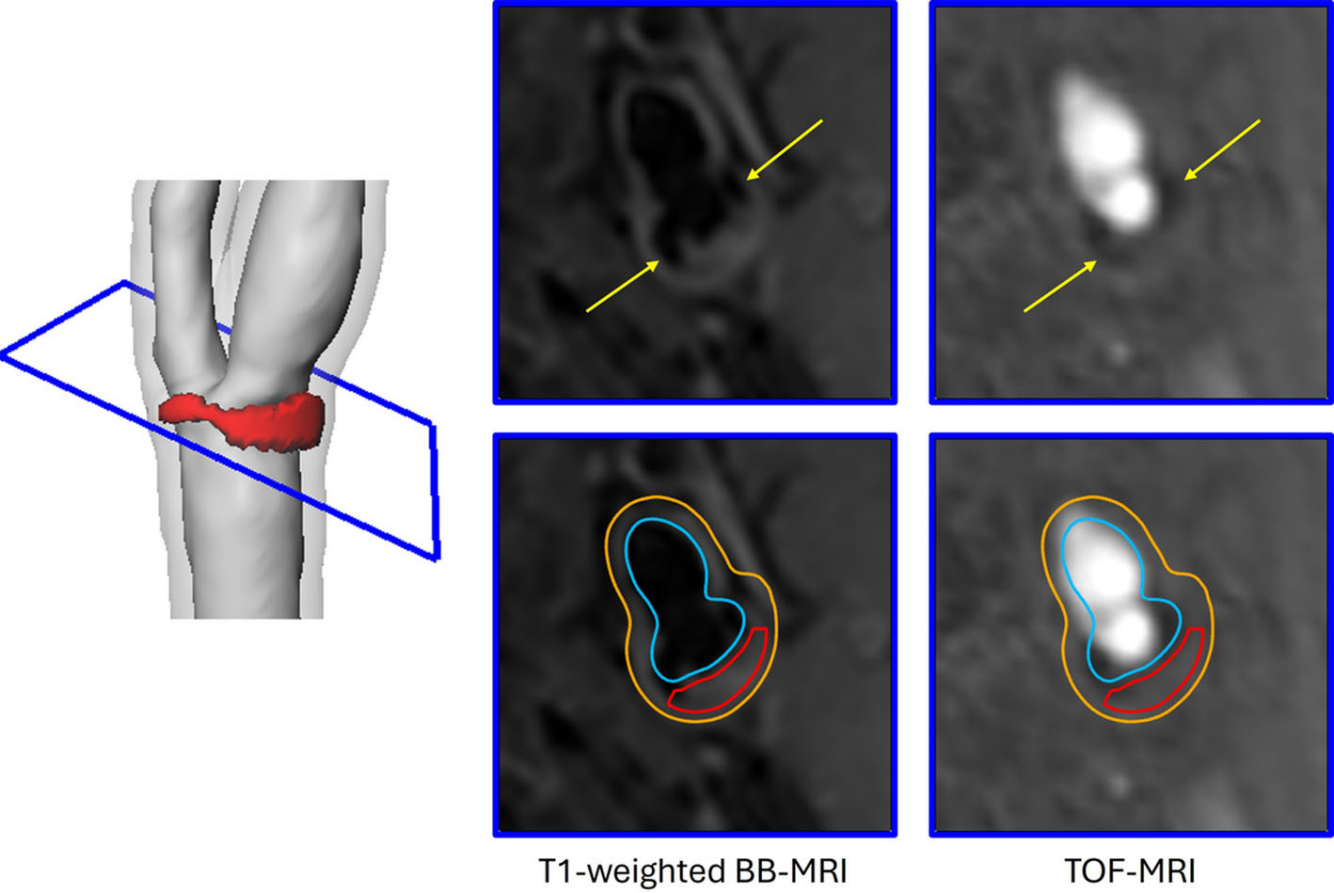
Cross section of the T1-weighted BB-MRI and TOF-MRI of the follow-up examination in Fig. 4c. The outer vessel wall (orange), inner vessel wall (blue), and lesion region (red) show that the 3D segmentation failed in this case, as the hypo-intense areas (yellow arrow) are not part of the lumen but are calcifications and part of the vessel wall and lesion

The proposed method is highly dependent on the 3D segmentation of the artery wall. In Fig. 4c, d the follow-up examination shows a lesion volume decrease for the follow-up examination. Figure 7 shows a T1-weighted BB-MRI and a Time-of-Flight MRI (TOF-MRI) of the follow-up examination and clearly shows that the underlying automatic lumen segmentation was not correct. The hypo-intense areas close to the lumen (yellow arrow) are calcification, as they also appear hypo-intense in the TOF-MRI. This example highlights that an improved segmentation method that registers and segments multi-contrast MRI would improve the reliability of the lesion extraction.

***Visual assessment of lesion shape and vessel wall thickness***

The BB-MRI datasets contain the carotid artery in the bifurcation area, and the underlying graph is a tree of depth one. Previous methods for the creation of vessel maps addressed this with different strategies to cut the surface of the artery [16, 17, 19]. We took a different approach and only created a vessel wall map for the regions with increased vessel wall thickness. In the taxonomy for vessel maps [27], this means we use a graph segment, precisely a part of a segment, as input graph. We use a projection layout and a mesh parametrization for the geometric mapping. The VWT is color-coded on the map. By doing so we want to improve the assessment of the lesion morphology. Comparing Fig. 4c and e the clear difference between a small area of vessel wall thickening and a wider area of vessel wall thickening can be easily seen. The visualization allows a comparison between the lesion shape of the baseline and follow-up examinations, but the vessel maps do not follow a standardized layout and a point-to-point correspondence between baseline and follow-up is not possible. This could be enabled by the more standardized approach proposed by Choi et. al. [17]. The unfolding depends on the lesion mesh extraction and can be misleading if this step fails. This approach might be applicable to lesions in other arteries, but we did not test it. The approach tries to preserve the area as it uses the as-rigid-as-possible method.

***Quantitative assessment of lesions***

Previous studies estimated plaque volume by manually or semi-automatically segmenting the entire carotid wall, using wall volume as a proxy for plaque progression [10, 34]. This approach captures total wall volume rather than actual plaque volume and relies on manual input. In contrast, our method computes the volume of the area with increased VWT, providing a closer approximation of true plaque volume. The relevance of the vessel volume for stroke risk assessment has been shown before [9, 10, 34], but further studies are needed to determine the influence of the parameters lesion volume, area, and compactness. We observed that most lesions exhibit small volumes, typically less than $50\,{\text {mm}}^{3}$, but the method was capable of extracting plaques with higher volumes up to $996.9\,{\text {mm}}^{3}$.

We analyzed signal intensities within the lesion mesh and found that different components can be distinguished in the resulting histograms. These histograms, representing the complete lesion, are independent of cross-sectional placement and provide an overview of the intensity distribution. However, we believe that reliable classification of plaque types and composition is not feasible using only T1-weighted BB-MRI. This would require extending the segmentation and lesion extraction to include the multi-contrast imaging approach used by Cai et al. [3].

***Applicability***

The method extracts lesions of varying sizes, at different positions, and of different shapes. The presented processing pipeline includes separate modules for segmentation and quantitative analysis, which can be adapted to enable lesion extraction and analysis in other tomographic imaging modalities and vessel systems. If a 3D vessel wall segmentation exists, only the lesion threshold *lt* has to be adapted.

## Limitations

The lesion extraction is only based on the distance encoding from 3D vessel wall segmentation, making it sensitive to segmentation quality. In our experiments, segmentation was based only on T1-weighted BB-MRI, which may lead to lesion underestimation if calcifications are wrongly segmented as lumen. Additionally, the method cannot extract a lesion if the thickness of the vessel wall is not increased, missing lesions that are only showing as intensity changes. The current mesh unfolding method does not perform a cutting and therefore cannot visualize lesions that encircle the entire vessel.

## Conclusion and future work

In conclusion, this method provides a quantification of the region with increased VWT and allows an analysis of the intensity distribution within the atherosclerotic lesion. Enhancements in vessel wall segmentation can be integrated into the existing pipeline to improve method accuracy. Further investigation is required to assess the clinical utility of the quantitative parameters for atherosclerosis progression monitoring and risk prediction. A promising direction for future work is extending the method to incorporate multi-contrast MRI, which may improve the vessel wall segmentation and enable automatic classification of atherosclerotic carotid lesions.
